# Attenuated Porcine Reproductive and Respiratory Syndrome Virus Regains Its Fatal Virulence by Serial Passaging in Pigs or Porcine Alveolar Macrophages To Increase Its Adaptation to Target Cells

**DOI:** 10.1128/spectrum.03084-22

**Published:** 2022-10-11

**Authors:** Jingjing Wang, Mengsi Zhang, Xiaochun Cui, Xiang Gao, Weifeng Sun, Xinna Ge, Yongning Zhang, Xin Guo, Jun Han, Lei Zhou, Hanchun Yang

**Affiliations:** a Key Laboratory of Animal Epidemiology of Ministry of Agriculture and Rural Affairs, College of Veterinary Medicine, China Agricultural University, Beijing, People’s Republic of China; Changchun Veterinary Research Institute

**Keywords:** porcine reproductive and respiratory syndrome virus, attenuated strain, reverse passage, virulence reversion, replication fitness, whole genome, mutation analysis

## Abstract

Porcine reproductive and respiratory syndrome (PRRS) is a globally important disease threatening the pork industry, and modified live-virus (MLV) vaccines are widely used for its prevention. However, PRRS MLV shows high potential for reversion to virulence, leading to a major concern about its safety. Yet the revertant mechanism is still poorly understood. Here, attenuated virus JXwn06-P80, derived from the highly pathogenic PRRS virus (PRRSV) strain JXwn06 by serial passaging in MARC-145 cells, was reversely passaged in pigs through intranasal inoculation to mimic natural infection for 13 rounds, and the pathogenicity of viruses at the 3rd, 5th, 9th, 10th, and 11th passages was evaluated in pigs. From the 9th passage, the viruses caused mortality, which was related to their increased adaptability and replication efficiency (100 times higher than those of JXwn06-P80) in porcine alveolar macrophage (PAM) target cells. Similarly, JXwn06-P80 could also regain fatal virulence through reverse passage in PAMs for 25 or more passages, indicating that the increased adaptability in PAMs directly contributes to its regained fatal virulence. Next, the full-genome sequences were analyzed to explore the genetic evolutionary processes during adaptation both *in vivo* and *in vitro*. Finally, by a reverse genetic operation, four reverse mutation sites, NSP12-W121R, ORF2b (open reading frame 2b)-H9D, ORF5-H15L, and ORF5-V189L, were finally identified to partially contribute to the ability of the virus to adapt to PAMs, which may be related to virulence reversion during reverse passage. These findings provided direct scientific evidence for the virulence reversion of PRRS MLV and provided valuable clues for exploring its molecular mechanism.

**IMPORTANCE** Reversion to virulence of a live attenuated vaccine is a public concern; however, direct scientific evidence is limited, and the mechanism is still poorly understood. Here, we present direct evidence for the reversion to virulence of PRRS MLV after serial passaging in pigs or target cells and found a correlation between virulence reversion and increased replication fitness in primary PAMs. The genetic evolutionary process during adaptation will provide valuable clues for exploring the molecular mechanism of PRRS MLV virulence reversion and offer important implications for understanding the reversion mechanisms of other vaccines.

## INTRODUCTION

Porcine reproductive and respiratory syndrome (PRRS), characterized as reproductive failure in sows and respiratory disease in pigs of all ages, was first reported as a “mystery disease” in the United States in 1987, and later, similar clinical symptoms were also found in Germany in 1990 (1–3). Currently, this emerging and reemerging infectious disease can still cause serious economic losses and greatly disturb the global swine industry. Especially, many unparalleled outbreaks of atypical PRRS, caused by variant strains with increased fatal pathogenicity, have been continuously reported in China, Eastern Europe, and the United States, which have deepened the awareness of the impact of PRRS on herd health (4–6). The causative agent, PRRS virus (PRRSV), which was first identified in 1991 (7), is an enveloped, positive-sense single-stranded RNA (+ssRNA) virus. It is newly classified into the genus *Porartevirus* of the family *Arteriviridae* of the order *Nidovirales* (8, 9), and there are two genotypes of PRRSV with obvious genetic and antigenic differences, represented by two prototype strains: Lelystad virus (LV) (type 1 or European type) and VR-2332 (type 2 or North American type) (9). The PRRSV genome is approximately 15 kb long, with at least 12 identified open reading frames (ORFs). ORF1a and ORF1b occupy more than two-thirds of the genome at the 5′ terminus and encode the polyproteins pp1a and pp1ab, which are further processed into 16 nonstructural proteins (NSPs) responsible for viral transcription and replication. The remaining one-third of the genome at the 3′ terminus encodes the viral structural proteins, which are involved in receptor binding, virus entry, and neutralizing antibody elicitation, etc., and they are translated from a set of subgenomic RNAs (sgRNAs), individually (10). PRRSV is an RNA virus showing extensive genetic and antigenic diversity among field strains, attributed to its low replication fidelity and strain recombination (11, 12), and selection pressure from the host can further drive PRRSV evolution, which may be related to its virulence changing or escaping the host immune responses (10, 13, 14).

PRRSV preferentially infects monocyte/macrophage lineages. Especially, porcine alveolar macrophages (PAMs) are identified as its principal target cells. Macrophages are involved in the detection, phagocytosis, and destruction of bacteria and other harmful organisms. Additionally, they are also recognized for their antigen-presenting, immunoregulatory, and effector functions in the specific immune response (15). PRRSV infection can induce dysfunction of macrophages, such as reductions in antigen phagocytosis or even cell death by necrosis and indirect bystander apoptosis (16–18). Also, PRRSV markedly alters the innate immune response and inflammatory and immunoregulatory cytokine secretion in infected macrophages (11, 19). So the characteristics of PRRSV infection and replication in host macrophages might influence their pathogenicity in a strain-specific manner.

A vaccine is an important tool for preventing and controlling infectious diseases in livestock, which may effectively reduce the risk of outbreaks and limit the transmission of pathogens in herds. Since the first commercial PRRS vaccine, a modified live virus (MLV), was launched in the United States in 1994, the PRRS MLV has been widely used for almost 30 years (20). Live attenuated vaccine strains can infect host animals, causing only mild or slight illness, but importantly, they can also produce viral antigens to induce the immune response of the host just like the wild-type (WT) parental viruses. As MLV can still replicate in the host’s immune cells, it has the potential for reversion to virulence and recombination with field strains, which significantly increases concerns about its safety (20). Furthermore, the commercially available PRRS MLV cannot offer sterilizing immunity against heterologous field strains that may promote PRRSV mutations to adapt to the host and confer escape from the host immune response. In PRRS MLV-vaccinated herds, pigs can develop viremia for up to 4 weeks postimmunization, increasing the possibility of shedding the vaccine virus to naive animals (21, 22). Currently, numerous field strains with molecular markers of PRRS MLV strains, such as nucleotide sequences identical to that of the vaccine strain, have been frequently isolated in the field (21, 22). Meanwhile, the high reversion potential of highly pathogenic PRRSV (HP-PRRSV)-derived vaccine candidates was also confirmed in our previous study (23). However, there is little direct scientific evidence for a virulence reversion event, and the mechanism of PRRSV reversion to virulence is still poorly understood. Exploring the evolutionary process that leads to an attenuated vaccine strain changing into a circulating and pathogenic form is important for evaluating the risks of current vaccination strategies and designing safer vaccines.

In this study, a highly attenuated PRRSV strain, JXwn06-P80, derived from HP-PRRSV JXwn06, was reversely passaged in both pigs and PAM target cells under laboratory conditions, and the pathogenicity and genomic sequences of revertant strains were analyzed after the attenuated strain regained its remarkable fatal virulence.

## RESULTS

### An attenuated PRRSV strain can regain its fatal virulence via serial passaging in pigs.

To confirm if the highly attenuated PRRSV strain JXwn06-P80 has a high potential to regain its fatal virulence when it is continuously circulating in herds, the attenuated virus JXwn06-P80 was serially passaged in pigs through intranasal inoculation to mimic natural PRRSV infection in herds, and the pathogenicity of the virus at 5 different representative passages (PIG-R-3, PIG-R-5, PIG-R-9, PIG-R-10, and PIG-R-11) was evaluated in pigs and compared with that of strain JXwn06-P80.

**FIG 1:**
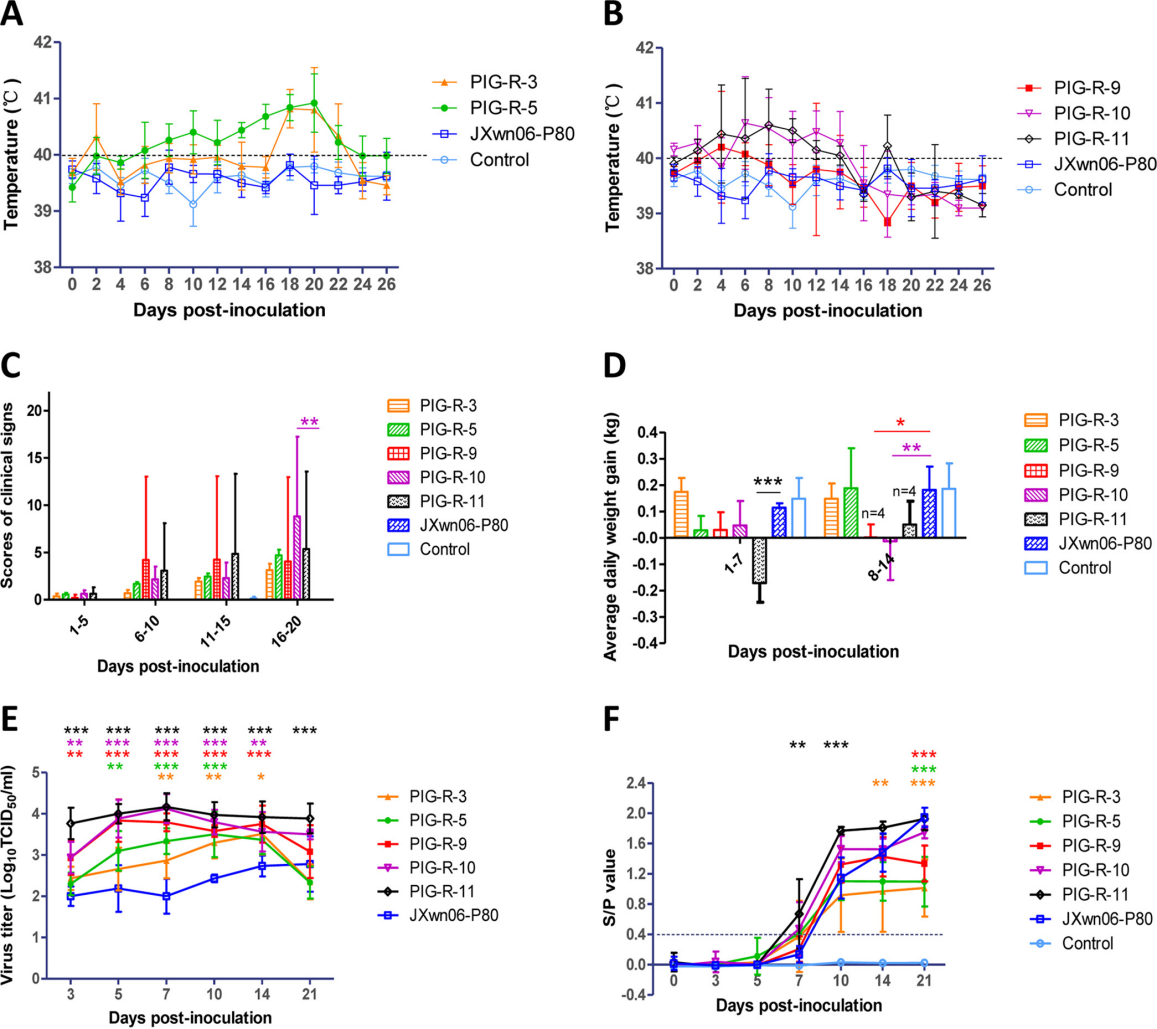
Clinical scores, viremia, and PRRSV-specific antibody kinetics of pigs inoculated with different PRRSV strains passaged *in vivo*. (A to D) Rectal temperatures (A and B), average clinical scores (C), and average daily weight gains (D) of the inoculated pigs. Clinical scoring consisted of the gross clinical score (GCS), respiratory clinical score (RCS), and nervous sign score (NSS). Total scores for each pig were the sum of the GCS, RCS, and NSS values, ranging from 0 to 15. A score of 20 was given when a pig died. (E) Viral loads in the sera of inoculated pigs on different days after inoculation. Virus titers were determined by a microtitration infectivity assay in primary PAMs. (F) The PRRSV-specific antibody kinetics of pigs were detected by using the Idexx PRRS X3 enzyme-linked immunosorbent assay (ELISA) kit. The antibody level is expressed as the sample value/positive value (S/P) ratio, and a ratio of ≥0.4 is regarded as seroconversion. The data are shown as means ± SD (error bars). Statistical differences are labeled according to two-way ANOVA followed by a Bonferroni posttest. Asterisks of different colors (according to the colors of lines or bars for that group in the key) indicate a significant difference between the *in vivo*-passaged virus group and the JXwn06-P80 group for the average clinical scores, ADWGs, viral loads, or S/P values (*, *P < *0.05; **, *P < *0.01; ***, *P < *0.001).

The body temperatures of pigs in the JXwn06-P80 and control groups remained normal throughout the experiment, but the rest of the pigs inoculated with reversely passaged virus showed fever, with increased mean rectal temperatures of above 40°C for more than 4 days at least, and the highest body temperature of individual animals reached 41.9°C in the PIG-R-11 group (Fig. 1A and B). The elevated temperature coincided with severe clinical respiratory disease, which was similar to that of wild-type JXwn06-inoculated pigs, including depression, anorexia, lethargy, rubefaction, respiratory distress, shivering, and paralysis; however, the onset was later (6 to 10 days postinoculation [dpi]) and relatively weaker than that in our previous animal studies (23). Generally, the pigs in the PIG-R-9, PIG-R-10, and PIG-R-11 groups showed higher scores for clinical signs than those in the PIG-R-3 and PIG-R-5 groups (Fig. 1C). The data on average daily weight gain (ADWG) were also consistent with this trend, in that the ADWGs of the PIG-R-3 and PIG-R-5 groups were similar to that of the JXwn06-P80 group; meanwhile, PIG-R-9, PIG-R-10, and PIG-R-11 inoculation significantly reduced the ADWG compared to inoculation with JXwn06-P80 (Fig. 1D).

Viremia could be detected in all PRRSV-inoculated groups, and the viral loads in the reversely passaged virus groups were significantly higher than that in the JXwn06-P80 group at almost all time points. The highest viral titer could reach above 10^4.10^ tissue culture infective doses (TCID_50_)/mL in the PIG-R-11 group, and the viremia data indicated that the ability of PRRSV to adapt to the host was continuously increased during serial passage in pigs (Fig. 1E). The serology analysis showed that all reversely passaged virus groups seroconverted slightly earlier than did the JXwn06-P80 group, with a higher sample value/positive value ratio (S/P ratio) before 10 dpi, and the pigs in the PIG-R-11 group showed the highest mean S/P ratios at all time points (Fig. 1F). Meanwhile, all pigs in the control group remained serologically PRRSV negative during the whole period.

**FIG 2:**
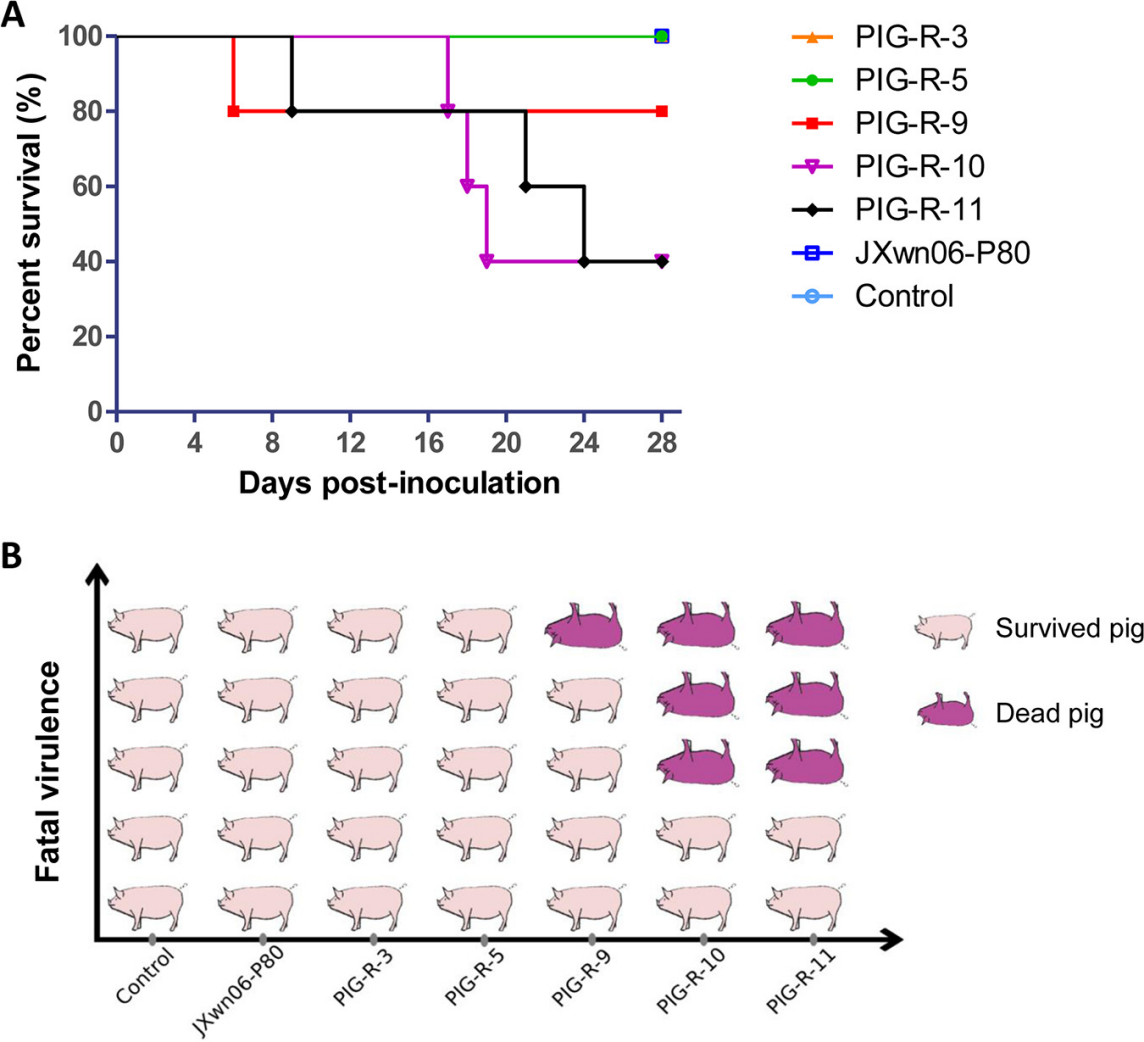
Survival curves of pigs inoculated with different PRRSV strains passaged *in vivo*. The time of pig death (A) and mortality (B) for each group are shown.

In this 28-day-long animal study, mortality was observed in the PIG-R-9, PIG-R-10, and PIG-R-11 groups (with 1/5, 3/5, and 3/5 pigs dying, respectively). Pig death occurred as early as 6 days after inoculation in the PIG-R-9 group, and the last death was observed in the PIG-R-11 group at 24 dpi. All pigs in the PIG-R-3, PIG-R-5, JXwn06-P80, and control groups survived during the whole experimental period (Fig. 2A and B). The survival curve shown in Fig. 2A directly indicated that the attenuated JXwn06-P80 strain can regain fatal virulence through reverse passage in its host.

### Serially passaged viruses caused severe gross and microscopic lesions.

**FIG 3:**
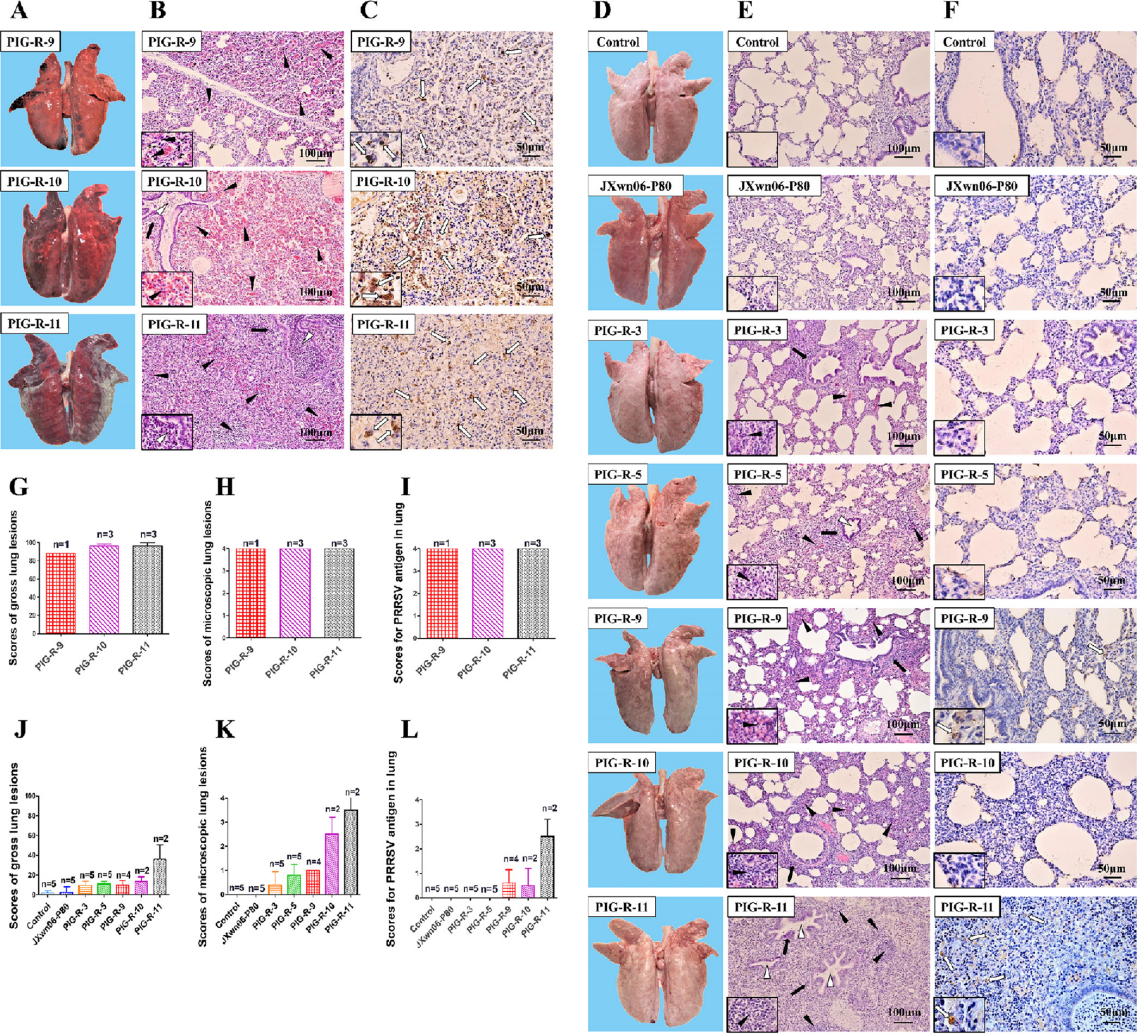
Lung lesions and immunohistochemistry examinations of pigs inoculated with different PRRSV strains passaged *in vivo*. (A, D, G, and J) Representative pictures of gross lung lesions and average scores of gross lung lesions from dead pigs during the experiment (A and G) and euthanized pigs at the end of the experiment (D and J). Gross lung lesions were graded based on the percentage of the lung area affected by pneumonia. (B, E, H, and K) Microscopic lung lesions stained with hematoxylin and eosin (H&E) and average microscopic lung lesion scores from dead pigs during the experiment (B and H) and euthanized pigs at the end of the experiment (E and K). Microscopic lesions were scored based on the severity of interstitial pneumonia. Solid arrows indicate thickening of the interlobular septa or infiltration of inflammatory cells around the bronchioles. Hollow triangles indicate inflammatory cells, necrotic debris, and exfoliated epithelial cells that infiltrated the bronchioles. Solid triangles indicate hemorrhage or infiltration of inflammatory cells within alveolar septa and alveolar spaces. (C, F, I, and L) Immunohistochemistry (IHC) examinations and average scores for PRRSV antigen in the lungs of dead pigs during the experiment (C and I) and euthanized pigs at the end of the experiment (F and L). A monoclonal antibody (SDOW17) specific for the N protein of PRRSV was used for IHC examination. The macrophages stained intensely dark brown, representing PRRSV antigen-positive cells, and the numbers of positive cells in the lungs were scored. Hollow arrows indicate positive signals in macrophages within or around the alveolus and bronchus.

Necropsy and gross lung lesion examinations were immediately performed once the inoculated pigs died during the experiment. All dead pigs in PIG-R-9-, PIG-R-10-, or PIG-R11-infected groups presented severe interstitial pneumonia with extensive and marked pulmonary edema, hemorrhage, and consolidation (Fig. 3A), with scores of gross lung lesions of >85 (Fig. 3G). By the end of the experiment, the surviving pigs were also euthanized for necropsy. Slight gross lung lesions were observed in the pigs from the PIG-R-3 and PIG-R-5 groups; meanwhile, mild lesions with several small pulmonary edemas and consolidations, distributed at the cranial and middle lobes, could be observed in PIG-R-10- and PIG-R11-infected pigs. Most JXwn06-P80-infected pigs and all pigs in the control group showed no obvious interstitial pneumonia (Fig. 3D and J).

Microscopic lung lesions of the inoculated pigs were further observed after hematoxylin and eosin (H&E) staining, and the PRRSV antigen distribution in the lungs was examined by immunohistochemistry (IHC) analysis with PRRSV N-specific monoclonal antibody (mAb). The lungs of the pigs that died during the experiment in the PIG-R-9-, PIG-R-10-, and PIG-R11-infected groups exhibited severe histopathological changes characterized by the complete (PIG-R-10 and PIG-R11) or extensive (PIG-R-9) disappearance of lung structure, hemorrhage, thickening of the interlobular septa, and infiltration of inflammatory cells and necrotic debris into both the alveolar spaces and bronchioles (Fig. 3B). Immunohistochemical staining showed that the lungs of these dead pigs were filled with PRRSV-positive signals, which were generally located in the alveolar and septal macrophages around the bronchia, bronchioles, and alveolar septa (Fig. 3C). The mean histopathological and immunohistochemical scores of these groups could even reach 4, with no obvious differences among them (Fig. 3H and I). For the surviving pigs, the lungs of PIG-R-11-infected pigs showed histopathological changes similar to those of the dead ones, and the rest of the pigs in the PIG-R-3, PIG-R-5, PIG-R-9, and PIG-R-10 groups presented moderate microscopic lesions with a partial disappearance of lung structure, minor hemorrhage, and inflammatory cells and necrotic debris within both the alveolar spaces and bronchioles. Meanwhile, only very slight histopathological changes were found in the JXwn06-P80 group (Fig. 3E and K). For the surviving pigs, PRRSV-positive signals were found mainly in PIG-R-11-infected pigs, and only sporadic positive signals were observed in the PIG-R-9 and PIG-R-10 groups (Fig. 3F and L).

### The *in vivo* reversely passaged PRRSV strains displayed increased replication efficiencies in primary PAMs.

**FIG 4:**
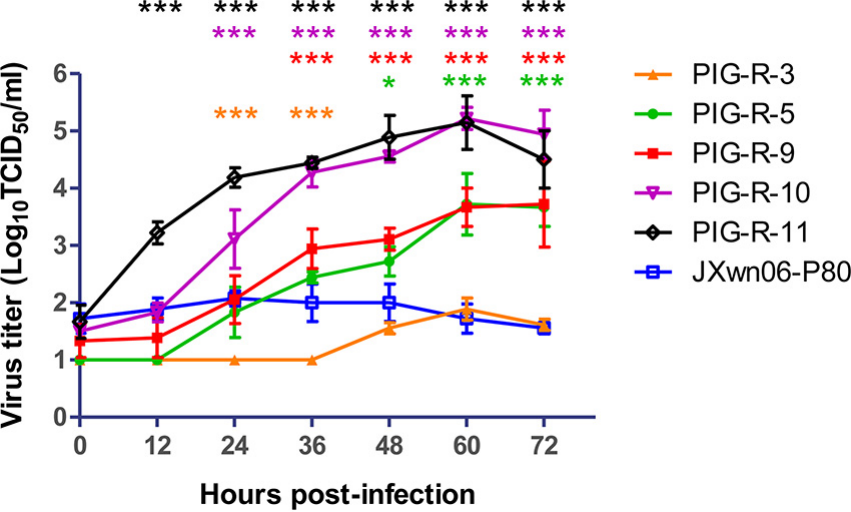
Growth kinetics of the *in vivo* reversely passaged PRRSV strains in primary PAMs. PIG-R-3-, PIG-R-5-, PIG-R-9-, PIG-R-10-, PIG-R-11-, and JXwn06-P80-infected primary PAMs at an MOI of 0.01 are shown. Virus titers were determined by microtitration infectivity assays in primary PAMs. The data are shown as means ± standard deviations (SD) (error bars) from three independent experiments. Statistical differences are labeled according to two-way ANOVA followed by a Bonferroni posttest. Asterisks of different colors indicate a significant difference between the *in vivo*-passaged virus group and the JXwn06-P80 group (*, *P < *0.05; ***, *P < *0.001).

The animal inoculation study indicated that reversely passaging the attenuated HP-PRRSV in its host could regain its fatal virulence, with increased viremia and viral distribution in the lungs. To test the hypothesis that *in vivo*-passaged viruses obtain improved replication capabilities in their primary PAM host cells, the growth kinetics of these viruses on primary PAMs were further investigated *in vitro* and compared with those of JXwn06-P80. The multistep growth curves showed that JXwn06-P80 had only limited growth in primary PAMs, with peak viral titers below 10^2.10^ TCID_50_/mL, and except for strain PIG-R-3, all reversely passaged viruses showed robust replication capabilities. Especially, the titers of strains PIG-R-10 and PIG-R-11 were significantly higher than that of JXwn06-P80 at almost all time points, with peak viral titers above 10^5.20^ TCID_50_/mL (Fig. 4). These results suggested that reversely passaging the attenuated HP-PRRSV in pigs could progressively increase its adaptability and replication efficiency in its target host cells, which might contribute to increased pathogenicity.

### The *in vitro* reversely passaged PRRSV strains showed improved replication efficiencies in primary PAMs.

**FIG 5:**
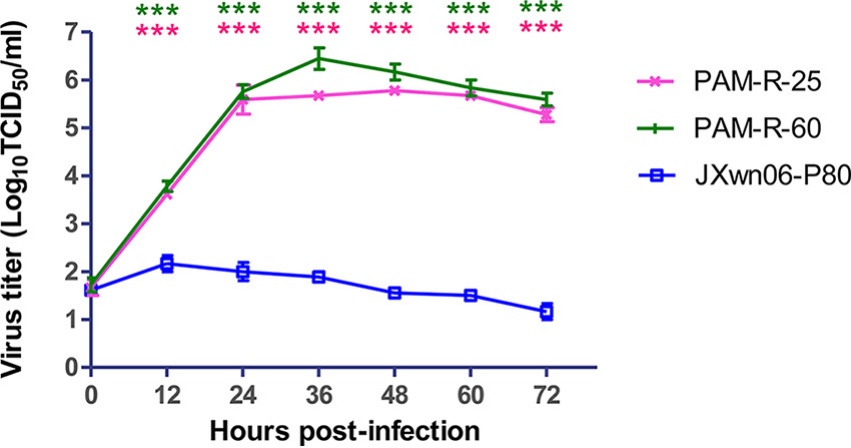
Growth kinetics of the *in vitro* reversely passaged PRRSV strains in primary PAMs. PAM-R-25-, PAM-R-60-, and JXwn06-P80-infected primary PAMs at an MOI of 0.01 are shown. Virus titers were determined by microtitration infectivity assays in primary PAMs. The data are shown as means ± SD (error bars) from three independent experiments. Statistical differences are labeled according to two-way ANOVA followed by a Bonferroni posttest. Asterisks of different colors indicate a significant difference between the *in vivo*-passaged virus group and the JXwn06-P80 group (***, *P < *0.001).

To further identify if the increased adaptation to primary PAMs is directly related to its fatal pathogenicity to pigs, the attenuated JXwn06-P80 virus was reversely passaged *in vitro* for 60 rounds in its PAM target cells. The viruses at passage 25 (P25) and P60, named PAM-R-25 and PAM-R-60, were first selected to evaluate their growth characteristics on primary PAMs. The multistep growth curve showed that PAM-R-25 and PAM-R-60 presented significantly higher titers than those of JXwn06-P80 at all time points, with peak viral titers being close to 10^6.20^ TCID_50_/mL and above 10^6.40^ TCID_50_/mL, respectively (Fig. 5). These data suggest that reversely passaging the attenuated HP-PRRSV in primary PAMs could significantly improve its PAM adaptability.

### The *in vitro* reversely passaged PRRSV strains displayed increased replication capabilities and fatal virulence in pigs.

To further investigate if the improved PAM adaptability could directly contribute to PRRSV fatal pathogenicity, the viruses PAM-R-25 and PAM-R-60 were submitted to another batch of animal trials. The processes, including animal ages, numbers of animals in each group, viral inoculation doses and pathways, and all clinical parameters investigated in this trial, were the same as those of the first one described above. Additionally, the wild-type parental strain JXwn06 (P8) was also used as a positive control here.

**FIG 6:**
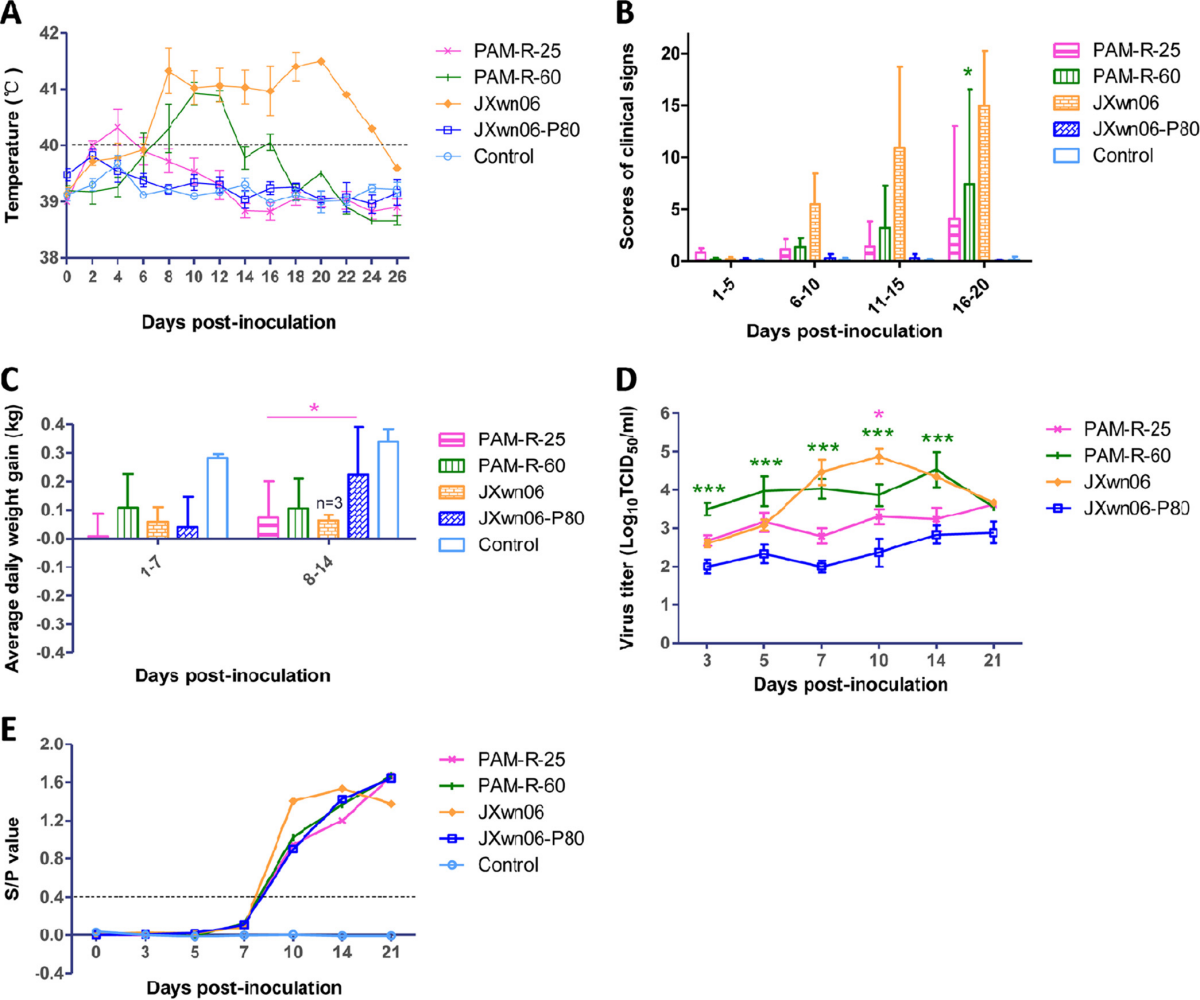
Clinical scores, viremia, and PRRSV-specific antibody kinetics of pigs inoculated with different PRRSV strains passaged *in vitro*. (A to C) Rectal temperatures (A), average clinical scores (B), and average daily weight gains (C) of the inoculated pigs. (D) Serum viral loads in pigs inoculated with the *in vitro* reversely passaged PRRSV strains. Virus titers were determined by a microtitration infectivity assay in primary PAMs. (E) Serum antibody kinetics of pigs in different groups. The data are shown as the means ± standard deviations (SD) (error bars), except for only one pig left in the JXwn06-infected group at 21 dpi. Asterisks of different colors indicate a significant difference between the *in vitro*-passaged virus group and the JXwn06-P80 group for the average clinical scores, ADWGs, viral loads, or S/P values (*, *P < *0.05; ***, *P < *0.001).

Pigs inoculated with the PAM-R-25 or PAM-R-60 virus began exhibiting fever within 2 to 6 dpi and maintained a mean renal temperature of >40°C for 4 days or 8 days, respectively. The peak temperature of the PAM-R-60 group was close to 41°C, significantly higher than that of the PAM-R-25 group (Fig. 6A). As in the study described above, the control and JXwn06-P80 groups did not show fever during the whole experimental period, and the JXwn06 group showed the highest body temperature and fever remaining for more than 18 days. Typical clinical signs of HP-PRRS, including depression, anorexia, lethargy, rubefaction, increased respiratory rates or even respiratory distress, shivering, and paralysis, were observed in both the PAM-R-25 and PAM-R-60 groups, especially in the pigs that died before the end of the trial. However, the severity of clinical parameters was still less than that in the JXwn06 group (Fig. 6B). The ADWG data showed that pigs in both the PAM-R-25 and PAM-R-60 groups grew more slowly than did those in the JXwn06-P80 group from 8 to 14 dpi, with a significant difference between the PAM-R-25 and JXwn06-P80 groups (Fig. 6C). The pigs in the control group remained clinically normal for the duration of the study.

The viremia of inoculated pigs was monitored, and the results showed that the viral loads of PAM-R-25 and PAM-R-60 were higher than that of JXwn06-P80, with a significant difference between PAM-R-60 and JXwn06-P80 at all time points, and the peak titer of PAM-R-60 reached 10^4.00^ TCID_50_/mL; however, it was still lower than that of JXwn06 (Fig. 6D). These results indicated that the reverse passaging of attenuated HP-PRRSV in primary PAMs can also increase its host adaptability and replication efficiency *in vivo*. Also, there were no obvious serological differences among the PAM-R-25, PAM-R-60, and JXwn06-P80 groups, and all inoculated pigs seroconverted at around 10 dpi (Fig. 6E).

**FIG 7:**
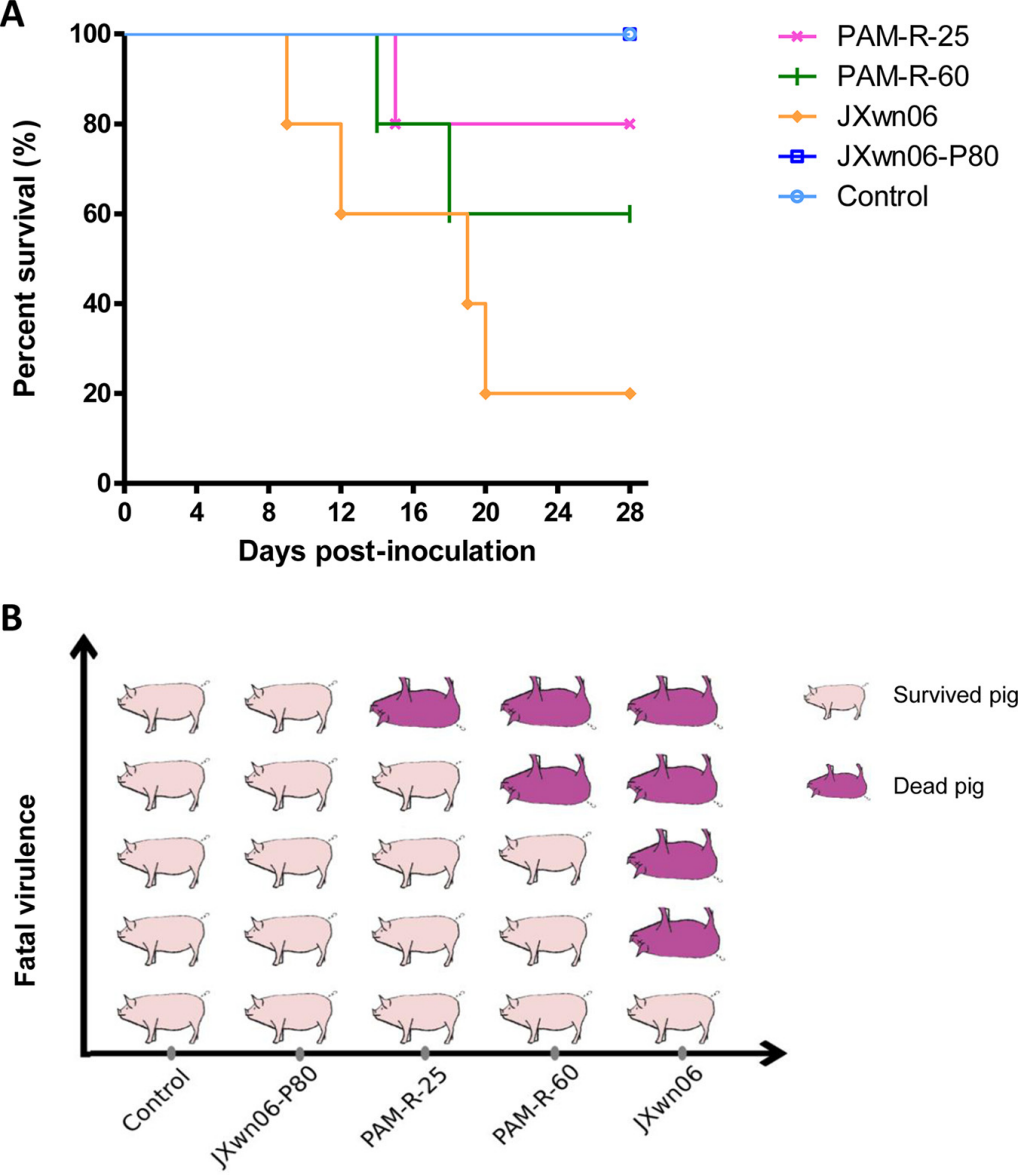
Survival curves of pigs inoculated with different PRRSV strains passaged *in vitro*. The time of pig death (A) and mortality (B) for each group are shown.

In this animal inoculation study, 1 pig from the PAM-R-25 group died at 15 dpi, 2 pigs from the PAM-R-60 group died at 14 and 18 dpi, and 4 of 5 pigs in the JXwn06 group died at 9 to 20 dpi (Fig. 7A and B). There was no mortality in the JXwn06-P80 and control groups for the duration of the experiment. These results suggested that attenuated HP-PRRSV could regain its fatal virulence through reverse passage in primary PAMs.

### PAM-R-25 and PAM-R-60 cause gross and microscopic lesions that are more intense than those caused by JXwn06-P80.

**FIG 8:**
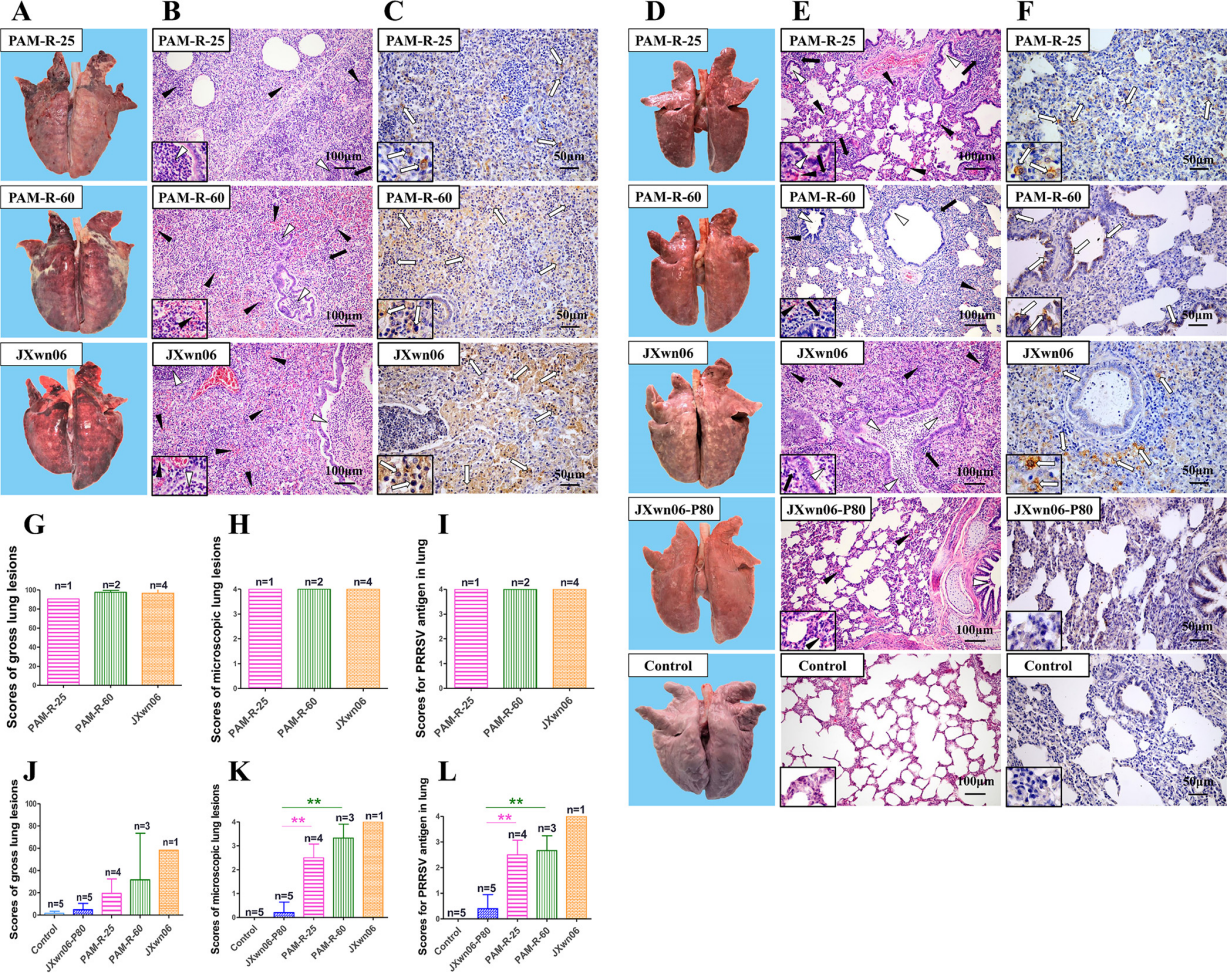
Lung lesions and immunohistochemistry examinations of pigs inoculated with different PRRSV strains passaged *in vitro*. (A, D, G, and J) Representative pictures of gross lung lesions and average scores of gross lung lesions from dead pigs during the experiment (A and G) and euthanized pigs at the end of the experiment (D and J). Gross lung lesions were graded based on the percentage of the lung area affected by pneumonia. (B, E, H, and K) Microscopic lung lesions stained with H&E and average microscopic lung lesion scores for dead pigs during the experiment (B and H) and euthanized pigs at the end of the experiment (E and K). Microscopic lesions were scored based on the severity of interstitial pneumonia. Solid arrows indicate thickening of the interlobular septa or infiltration of inflammatory cells around the bronchioles. Hollow triangles indicate inflammatory cells, necrotic debris, and exfoliated epithelial cells infiltrating the bronchioles. Solid triangles indicate hemorrhage or infiltration of inflammatory cells within alveolar septa and alveolar spaces. (C, F, I, and L) IHC examinations and mean scores for PRRSV antigen in the lungs of dead pigs during the experiment (C and I) and euthanized pigs at the end of the experiment (F and L) in each group. Hollow arrows indicate positive signals in macrophages within or around the alveolus and bronchus. Statistical differences are labeled according to a two-tailed untailed *t* test with Welch’s correction. Asterisks in different colors (according to the colors of lines or bars for that group in the key) indicate a significant difference between the *in vitro* reversely passaged virus-infected group and the JXwn06-P80-infected group (**, *P < *0.01).

As in the first animal experiment described above, gross/microscopic lung lesion and IHC analyses were carried out immediately after the pigs died or at the end of the experiment. Similar to JXwn06-infected pigs, all dead pigs in the PAM-R-60- or PAM-R-25-infected group presented severe interstitial pneumonia with extensive and marked pulmonary edema, hemorrhage, and consolidation (Fig. 8A). The gross lung lesion score of the dead pig in the PAM-R-25-infected group was slightly lower than those of pigs in the other two groups (Fig. 8G). Generally, the lung lesions were less severe for the euthanized pigs; moderate or mild interstitial pneumonia was observed in the JXwn06-, PAM-R-25-, and PAM-R-60-infected groups (Fig. 8D and J).

Microscopic lung lesions and PRRSV antigen distributions in lung tissues were also analyzed in the first animal trial, and the results suggested that the viruses PAM-R-25 and PAM-R-60 could cause similarly severe microscopic lung lesions and virus distributions in dead pigs (Fig. 8B, C, H, and I), and the overall severity of these parameters in euthanized pigs was significantly greater than that in the JXwn06-P80 group but still slightly less than that in the JXwn06 group (Fig. 8E, F, K, and L).

### Genomic changes in PRRSV during reverse passaging in pigs or PAMs.

To identify the genomic changes in PRRSV during reverse passaging in pigs or PAMs that might contribute to increased host adaptability or virulence, the whole-genome sequences of PRRSV strains PIG-R-3, PIG-R-5, PIG-R-9, PIG-R-10, PIG-R-11, PAM-R-25, and PAM-R-60 were obtained by reverse transcription-PCR (RT-PCR) and sequencing as described in our previous report (24). The assembled genomic sequences of these PRRSV strains were aligned with those of JXwn06 and JXwn06-P80, and the mutated sites are shown in Tables S1 and S2 in the supplemental material. Compared with JXwn06-P80, there were 10 to 17 amino acid mutations identified in the genomes of the viruses reversely passaged in pigs, among which 1 to 4 amino acid sites were reverse mutations, mutated back to the amino acids of the JXwn06 wild-type virus. Meanwhile, the numbers of mutated amino acids in PAM-R-25 and PAM-R-60 were 15 and 25, respectively; among them, 2 and 4 amino acid sites were mutated back to the sites in JXwn06 (Table 1). In general, the numbers of mutated nucleotides/amino acids increased with increasing passages (both *in vivo* and *in vitro*). Four sites, NSP7-L142P, NSP12-W121R, ORF3-S143F, and ORF5-V80G, were found to mutate back to the amino acid sites of JXwn06 during passaging in pigs, and all four reverse mutations fully changed back by passage 9 (PIG-R-9) (Table 2). Similarly, four reverse mutations, NSP12-W121R, ORF2b-H9D, ORF5-H15L, and ORF5-V189L, were found in the PRRSV genome during reverse passaging in primary PAMs (Table 3). Interestingly, the reverse mutation NSP12-W121R coexists in the viruses from both the *in vivo* and *in vitro* passaging studies.

**TABLE 1:** Statistics of mutations detected at different passages of PRRSV

PRRSV strain	No. of nt mutations^*a*^	No. of aa mutations^*a*^	No. of reversed nt mutations^*b*^	No. of reversed aa mutations^*b*^
PIG-R-3	23	10	2	1
PIG-R-5	26	14	6	3
PIG-R-9	27	13	6	4
PIG-R-10	37	16	6	4
PIG-R-11	36	17	6	4
PAM-R-25	23	15	3	2
PAM-R-60	34	25	5	4

a Quantities of nucleotide (nt) and amino acid (aa) mutations of the reversely passaged strains, compared with JXwn06-P80.

b Quantities of reversed nucleotides and amino acid mutations of the reversely passaged strains, compared with JXwn06-P80 and JXwn06.

**TABLE 2:** Reverse mutations in PRRSV passaged *in vivo*

PRRSV strain	Mutated site (nt/aa)^*a*^			
	NSP7 7121/142	NSP12 11880/121	ORF3 13033/143	ORF5 13936/80
JXwn06-P80	T/L	T/W	C/S	T/V
PIG-R-3	**C/P**	T/W	C/S	T/V
PIG-R-5	**C/P**	**C/R**	**T/F**	**C/A**
PIG-R-9	**C/P**	**C/R**	**T/F**	**G/G**
PIG-R-10	**C/P**	**C/R**	**T/F**	**G/G**
PIG-R-11	**C/P**	**C/R**	**T/F**	**G/G**
JXwn06	**C/P**	**C/R**	**T/F**	**G/G**

a The sites reversely mutated to the amino acids of JXwn06 are labeled in boldface type.

**TABLE 3:** Reverse mutations in PRRSV passaged *in vitro*

PRRSV strain	Mutated site (nt/aa)^*a*^			
	NSP12 11880/121	ORF2b 12012/9	ORF5 13741/15	ORF5 14262/189
JXwn06-P80	T/W	C/H	A/H	G/V
PAM-R-25	T/W	C/H	**T/L**	**T/L**
PAM-R-60	**C/R**	**G/D**	**T/L**	**T/L**
JXwn06	**C/R**	**G/D**	**T/L**	**T/L**

a The sites reversely mutated to the amino acid of JXwn06 are labeled in boldface type.

### The four reverse mutations in PAM-R-60 partially contributed to its adaptation to PAMs.

**FIG 9:**
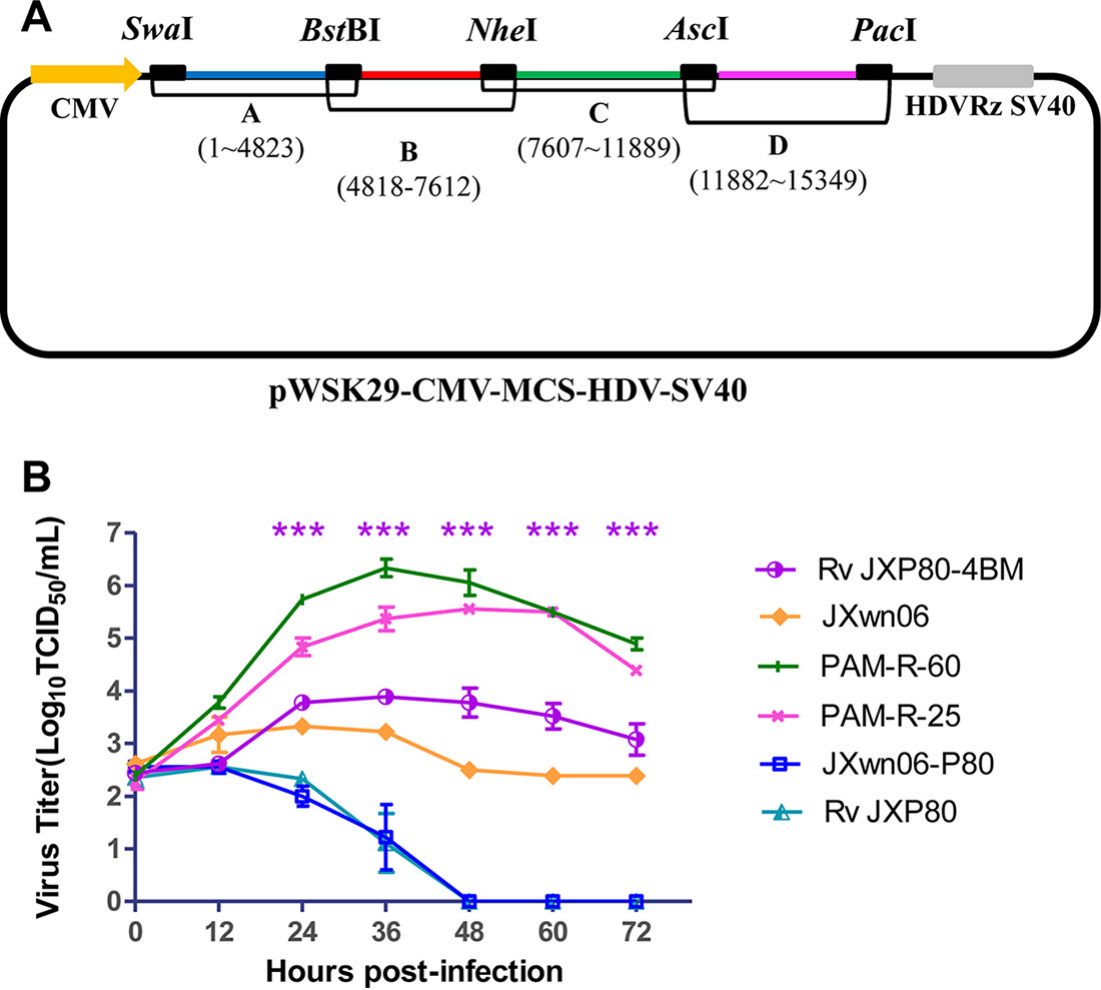
Growth kinetics of the rescued virus with reverse mutations in primary PAMs. (A) Strategy for the construction of the full-length cDNA clone of JXwn06-P80. Capital letters (A, B, C, and D) represent four fragments amplified from the JXwn06-P80 genome, with unique restriction enzyme cleavage sites in overlapping regions. SwaI was introduced into the 5′ end of fragment A, PacI was introduced into the 3′ end of fragment D, and BstBI was introduced between fragments A and B. Four fragments were assembled into the vector pWSK29-CMV-MCS-HDV-SV40 in the order D, C, B, and A. The full-length infectious clone plasmid was named pCMV-JXP80. HDVRz, hepatitis delta virus ribozyme. (B) Growth kinetics of the rescued virus with reverse mutations in primary PAMs. The rescued viruses or the attenuated JXwn06-P80 virus infected primary PAMs at an MOI of 0.01. Virus titers at different time points were determined by microtitration infectivity assays in primary PAMs. The data are shown as means ± SD (error bars) from three independent experiments. Statistical differences are labeled according to two-way ANOVA followed by a Bonferroni posttest. Asterisks indicate a significant difference in the virus titers between RvJXP80-4BM and JXwn06-P80 (***, *P < *0.001).

To identify if the four reverse mutation sites NSP12-W121R, ORF2b-H9D, ORF5-H15L, and ORF5-V189L were related to the increased PAM adaptability and even the fatal virulence of HP-PRRSV, the reverse genetic operation system was used to introduce these four mutation sites into the backbone of RvJXP80, which is the rescued virus from the full-length infectious clone plasmid of JXwn06-P80 (Fig. 9A). The rescued virus with the four reverse mutations NSP12-W121R, ORF2b-H9D, ORF5-H15L, and ORF5-V189L in the genome was named RvJXP80-4BM (4BM means four back-mutation sites). The growth kinetics of RvJXP80-4BM, PAM-R-25, PAM-R-60, RvJXP80, JXwn06-P80, and JXwn06 were analyzed in primary PAMs. The results showed that the titer of RvJXP80-4BM was significantly higher than those of RvJXP80 and JXwn06-P80 from 24 to 72 h postinoculation (hpi), but its titers were still lower than those of PAM-R-25, and PAM-R-60 (Fig. 9B). Interestingly, the titers of PAM-R-25, PAM-R-60, and RvJXP80-4BM were higher than those of the JXwn06 wild-type virus (Fig. 9B); however, the PRRSV-specific cytopathic effect (CPE) in JXwn06-inoculated PAMs was more severe, and more PAMs fell off from the bottom of the cell culture plate (data not shown).

A preliminary animal inoculation experiment with 3 specific-pathogen-free (SFP) pigs was first performed. The body temperatures (reaching 40°C at 2 to 4, 8, and 14 dpi), clinical signs, and gross and microscopic lung lesions of inoculated pigs all indicated that the pathogenicity of RvJXP80-4BM is slightly higher than that of JXwn06-P80; however, no mortality or severe clinical symptoms were observed (data not shown). These results indicated that these four reverse mutations might partially contribute to the adaptation of the virus to PAMs, but they are not enough to directly confer fatal virulence to the virus reversely passaged in primary PAMs. So the contribution of these mutated sites or the individual ones to PRRSV fatal virulence was not further investigated by the standard process described above.

## DISCUSSION

Just a few years after the initial outbreak of PRRS at the end of the 1980s, the first commercial PRRS vaccine was launched in the United States in 1994, which was attenuated through serial passaging on a monkey kidney cell line, MARC-145 (20). Until now, most commercial PRRSV MLV strains were commonly attenuated by repetitive passaging in cells, which were derived mainly from the monkey kidney MA104 cell line or BHK-21 cells with porcine CD163 receptor expression (20).

The PRRS MLV can induce a good protective immune response against homologous viruses, and can even partially protect against heterologous viruses, by lessening the disease in vaccinated pigs and reducing virus transmission in herds, which is regarded as a cost-effective way to reduce the impact of PRRS on farms. In the last 3 decades, almost 20 different MLV strains have been introduced to the global market (25); however, PRRS is still characterized by the difficulties of prevention and control. Except for the unsatisfying heterologous cross-protection, the safety issues of MLV are a major concern. Just like field strains, the PRRS MLV virus can still infect and replicate in a subset of monocyte-derived cells of the host, which raises the potential issues of reversion to virulence and recombination with field strains. The unnecessary massive vaccination with various PRRS MLV strains can cause the wide circulation of diverse vaccine strains or vaccine-derived strains in the field (26–30). Furthermore, reversion to virulence of HP-PRRS MLV candidate strains was also observed in our previous study (23). However, the molecular mechanism of these processes is still a knowledge gap, which is greatly hindering scientists aiming to design a better PRRS vaccine with improved stability and safety (25, 31, 32).

In China, all veterinary MLV candidates should pass an assessment of reversion to virulence in susceptible host animals for at least 5 passages, as a requirement of the safety evaluation to apply for registration by the Ministry of Agriculture and Rural Affairs. For PRRS MLV, these tests were carried out by the intramuscular inoculation of sera collected from pigs with viremia. However, viral transmission of MLV in the herd is usually through oral and nasal exposures. In this study, to mimic the natural transmission and adaptation processes of MLV strains under field conditions, intranasal inoculation was chosen for reversely passaging the virus in pigs. Besides, intranasal infection might provide higher selection pressure, which benefits screening out the viruses with better infectivity, adaptability, and replication efficiency from their quasispecies. Here, the highly attenuated PRRSV strain JXwn06-P80 was passaged in pigs for 13 rounds, resulting in reversion to virulence with symptoms typical of HP-PRRS and mortality in pigs, starting at the 9th passage of the virus.

In our previous study, two PRRSV vaccine candidates, JXM87 and JXM105, derived from serial passaging of the parental HP-PRRSV JX143 strain (GenBank accession no. EU708726), sharing high genetic similarities with strain JXwn06, were assessed for reversion to virulence through five back-passages in susceptible piglets, and increased clinical signs (mild) and lung lesions (significant) and decreased average daily weight gain were observed as important indicators of reversion to virulence (23). Compared with that study, the current study showed increased mortality, clinical signs, and lung lesions as well as decreased average daily weight gain (body weight loss). Besides, increases in viremia were observed in both studies (23).

Consistent with the results of our previous studies, the replication efficiency of the reversely passaged strains was significantly improved compared with that of JXwn06-P80 *in vivo*. Especially, the peak viral load of the PIG-R-11-inoculated group reached 10^4.00^ TCID_50_/mL, almost 100 times higher than that of the JXwn06-P80 group, and a trend toward host adaptability increasing with increasing passages can be found from the viremia data for infected pigs, which was also generally consistent with the severities of clinical parameters and the growth curves of viruses passaged on PAMs. So we wondered if the increased adaptation of attenuated HP-PRRSV to PAMs can directly contribute to reversion to fatal virulence. To confirm this hypothesis, the attenuated JXwn06-P80 strain was further reversely passaged in PAMs *in vitro* for 60 rounds, and the growth characteristics and pathogenicity of the viruses PAM-R-25 and PAM-R-60 were evaluated in both target cells (*in vitro*) and pigs (*in vivo*) using the same process. As a result, the *in vitro* reversely passaged strains can also revert to fatal virulence, causing symptoms typical of HP-PRRS, with fatal virulence for pigs. These results suggested that PRRSV replication abilities and adaptation to its target cells directly contribute to PRRSV reversion to virulence.

In the PRRSV research community, exploring the genetic determinants of virulence is always an attractive subject, which is regarded as the key point for exploring the mechanisms of virus attenuation and reversion to virulence. The mutation sites among different attenuated strains can be easily identified. However, the mechanism of attenuation is less known, and there are still many challenges and knowledge gaps on it (33, 34, 35). In previous studies, based on comparing the genomes of virulent parental/attenuated vaccine strains of PRRSV, it was identified only that multigenic determinants in both nonstructural and structural genes were associated with PRRSV virulence or attenuation (23). An et al. parallelly compared five pairs of parental wild-type/attenuated PRRSV strains, and no conserved mutation site was found among all of these attenuated strains (36). Moreover, by constructing chimeric viruses with a swapped fragment from the highly virulent strain FL12 and the attenuated vaccine strain PrimePac, Kwon et al. identified the NSP3 to -8 and GP5 proteins as the major virulence determinants and NSP1 to -3, NSP10 to -12, and GP2 as minor virulence determinants (37, 38). Similarly, our previous study found that NSP9 and NSP10, especially residues 586 and 592 of NSP9, are critical for determining the replication efficiency and fatal virulence of Chinese HP-PRRSV, which explained how PRRSV obtained its fatal virulence during evolution from earlier Chinese strains; however, in later studies, these mutation sites were not found to be related to the attenuation or reversion to virulence of HP-PRRSV-derived vaccines.

To further explore the mechanism of increased adaptation to target cells and reversion to virulence in this study, all genomic sequences of the PRRSV strains tested in the animal study were obtained to compare them with the sequences of the attenuated strain JXwn06-P80 and the original wild-type strain JXwn06. Four revertant amino acid mutations (NSP7-L142P, NSP12-W121R, ORF3-S143F, and ORF5-V80G) from *in vivo*-passaged viruses as well as another four revertant amino acid mutations (NSP12-W121R, ORF2b-H9D, ORF5-H15L, and ORF5-V189L) from *in vitro*-passaged viruses were identified. This is similar to the results of our previous study of JXM87 and JXM105, which have only five conserved mutations occurring in both strains, four of which cause revertant amino acid mutations (23). However, none of these mutations were found in the present study. Notably, in reverse passaging studies both *in vivo* and *in vitro*, reverse mutations were identified in the ORF5 coding region, which was also reported to be one of the major virulence determinants in previous studies, explored by swapping the fragment between HP-PRRSV and an attenuated vaccine strain or comparing the genomic sequences of five pairs of virulent parental/attenuated vaccine strains (36, 38). Another interesting mutation site is ORF2b-H9D, which is located at the E protein, a type I transmembrane protein consisting of a transmembrane domain (amino acids [aa] 29 to 47), an ectodomain (aa 1 to 28), and a cytoplasmic tail (aa 48 to 73) (36). The 9th amino acid residue of the E protein is a “hot spot” mutation site among many attenuated PRRSV strains and their corresponding parental WT viruses; for example, two HP-PRRSV-derived commercial MLV strains, JXA1-P80 and HuN4-F112, had the same D^9^-to-H^9^ mutation at their E proteins during serial passaging in MARC-145 cells (36, 39). Besides, the same mutation was also found in 2 pairs of virulent parental/attenuated vaccine strains, VR-2332/RespPRRS MLV and CH-1a/CH-1R. Furthermore, the same reverse mutation, ORF2b-H9D, was also found in the genomes of PRRSV NT1, NT2, and NT3, the virulent isolates reversed from MLV strain JXA1-P80 (40).

To confirm if these revertant mutations can directly contribute to PAM adaptation and the reobtained fatal virulence, we initially introduced the four revertant amino acid mutations (NSP12-W121R, ORF2b-H9D, ORF5-H15L, and ORF5-V189L from the strains reversely passaged in PAMs) into the genome of RvJXP80 via a reverse genetic operation. The rescued virus, RvJXP80-4BM, showed significantly higher viral titers in PAMs than those of its backbone and parental viruses RvJXP80 and JXwn06-P80, indicating that these four revertant amino acid mutations contribute to viral adaptation to PAMs. However, the titers of RvJXP80-4BM were not as high as those of PAM-R-25 and PAM-R-60 at all time points after inoculation, suggesting that other mutation sites may also be involved in the increased replication capabilities of the reversely passaged viruses in PAMs. Furthermore, the pathogenicity of RvJXP80-4BM has also been preliminarily evaluated in an inoculation pretest with 3 pigs, and the clinical symptoms and pathogenic lesions indicate that its pathogenicity is partially increased compared with JXwn06-P80. However, all pigs survived during the 28-day-long experiment, which suggested that only four revertant amino acid mutations were not enough to confer fatal virulence to the reversely passaged virus. So the formal pathogenicity study of RvJXP80-4BM with larger groups of animals was not performed, according to the 3R principle of laboratory animal usage. These data indicated that not only the four reverse mutation sites but also some other sites mutated during *in vitro* reverse passaging (see Table S2 in the supplemental material) may contribute to the adaptation to PAMs and the reobtained fatal virulence of the viruses. So positive selection site identification (41–43) and AlphaFold protein structure prediction (44, 45) might be used to further screen the candidate sites among the genomes in our future study.

### Conclusions.

In summary, a highly attenuated strain derived from HP-PRRSV regained its fatal virulence by serial passaging in pigs through intranasal inoculation or in primary PAMs, and the improved PAM adaptability of the reversely passaged viruses was identified to directly contribute to the regained high pathogenicity and reversion to fatal virulence. Also, the four reverse mutations NSP12-W121R, ORF2b-H9D, ORF5-H15L, and ORF5-V189L found in the reversely passaged strains in primary PAMs can partially contribute to the replication fitness in PAMs but were not enough to confer fatal virulence to the reversely passaged viruses. These findings provide a new clue for understanding the mechanism of reversion to virulence of the PRRS vaccine.

## MATERIALS AND METHODS

### Cells, viruses, and plasmids.

For PRRSV proliferation and titration, an MA104-derived monkey kidney cell line, MARC-145, was cultured in Dulbecco’s modified Eagle’s medium (DMEM) (Thermo Fisher Scientific) with 10% fetal bovine serum (FBS) (Gibco) and penicillin (50 U/mL)-streptomycin (50 μg/mL). Primary porcine alveolar macrophages (PAMs), the target cells of PRRSV, were prepared from 30-day-old specific-pathogen-free (SPF) pigs and cultured in RPMI 1640 medium (Thermo Fisher Scientific) with 10% FBS, as previously described (7, 46). All cells were cultured at 37°C in a humid 5% CO_2_ incubator. JXwn06-P80, an attenuated strain, was derived from HP-PRRSV JXwn06 (GenBank accession no. EF641008) by serial passaging in MARC-145 cells for 80 rounds and 3 rounds of purification by a plaque assay, as previously described (7, 46). Plasmid pWSK29-CMV-MCS-HDV-SV40, which contains a cytomegalovirus (CMV) promoter, a multiple-cloning site (MCS), a hepatitis delta virus (HDV) ribozyme, and a simian virus 40 (SV40) signal, was modified from the low-copy-number plasmid vector pWSK29 used previously for constructing PRRSV infectious clones in our laboratory (47).

### Virus titration.

All PRRSV titers were determined by a microtitration infectivity assay in primary PAMs. Briefly, confluent monolayers of primary PAMs cultured in 96-well plates were incubated with 10-fold serially diluted virus suspensions. After absorption for 2 h at 37°C with 5% CO_2_, the supernatant was removed and changed to RPMI 1640 medium supplemented with 2% FBS. The plates were incubated for an additional 60 h at 37°C with 5% CO_2_. Virus titers were determined and recorded as 50% tissue culture infective doses (TCID_50_) per milliliter according to the Reed-Muench method (48).

### Reversely passaging JXwn06-P80 in pigs.

For animal studies, SPF Large White pigs were purchased from the Beijing SPF Pig Breeding and Management Center and raised in the animal facilities of China Agricultural University (CAU).

To investigate if serially passaging the attenuated HP-PRRSV in its host animal can cause the strain to regain fatal virulence, a 35-day-old SPF pig was intranasally inoculated with JXwn06-P80 at a dose of 4 × 10^5.00^ TCID_50_, the inoculated pig was observed for 14 days, and it was then euthanized and necropsied on the 14th day postinoculation (dpi). Blood samples were collected at 3, 5, 7, 10, and 14 dpi to determine viremia, and the serum sample with the peak viral titer was further used to inoculate the next pig. During passages *in vivo*, 4 mL (passages 1 to 5) or 2 mL (passages 6 to 12) of serum with the peak viral titer was used to intranasally inoculate the next pig as described above. The PRRSV strains in the sera from the 3rd, 5th, 9th, 10th, and 11th passages were named PIG-R-3, PIG-R-5, PIG-R-9, PIG-R-10, and PIG-R-11, which were further selected for pathogenicity evaluation and sequence analysis.

### Reversely passaging JXwn06-P80 in primary PAMs.

To test if the attenuated PRRS MLV strain could regain fatal virulence by increasing its adaptation to primary PAMs, the virus JXwn06-P80 was also serially passaged in primary PAMs. Primary PAM monolayers in 6-well tissue culture plates were inoculated with the virus JXwn06-P80 at a multiplicity of infection (MOI) of 1. After 2 h of incubation at 37°C, the supernatant was removed, followed by washing with phosphate-buffered saline (PBS); the medium was changed to RPMI 1640 medium supplemented with 2% FBS; and the inoculated cells were then incubated at 37°C with 5% CO_2_. When approximately 75% of virus-infected primary PAMs exhibited cytopathic effect (CPE), the virus was harvested after freeze-thawing, and it was then further passaged. The 25th and 60th passages of the virus in this *in vitro* study, named PAM-R-25 and PAM-R-60, were further selected for pathogenicity evaluation and sequence analysis.

### Growth kinetics of reversely passaged viruses and rescued viruses.

To analyze the growth characteristics of the reversely passaged PRRSV strains and the rescued viruses, primary PAMs were inoculated with the *in vivo* reversely passaged strains (PIG-R-3, PIG-R-5, PIG-R-9, PIG-R-10, and PIG-R-11), the *in vitro* reversely passaged strains (PAM-R-25 and PAM-R-60), the rescued PRRSV strains (RvJXP80 and RvJXP80-4BM), or the control strains (JXwn06 and JXwn06-P80) at an MOI of 0.01. The titers of viruses at different time points, 0, 12, 24, 36, 48, 60, and 72 h postinoculation (hpi), were determined by using a microtitration infectivity assay in primary PAMs and expressed as TCID_50_ per milliliter as described above. The data on growth kinetics were collected from three completely independent repeats.

### Pathogenicity evaluation of the reversely passaged viruses.

Sixty 35-day-old SPF Large White pigs free of PRRSV, African swine fever virus (ASFV), porcine circovirus type 2 (PCV2), pseudorabies virus (PRV), classical swine fever virus (CSFV), and Mycoplasma hyopneumoniae were purchased as two batches (35 pigs for the first batch and the remaining 25 pigs for another batch) from the Beijing SPF Pig Breeding and Management Center and raised in the facility of CAU. The pigs were allowed to acclimate for 3 days before experiments.

To analyze the pathogenicity of the virus reversely passaged *in vivo*, 35 pigs were randomly divided into 7 groups (*n* = 5) and raised separately in different rooms. Pigs in each group were intranasally inoculated with 2 × 10^5.00^ TCID_50_ of virus (JXwn06-P80, PIG-R-3, PIG-R-5, PIG-R-9, PIG-R-10, or PIG-R-11) or 2 mL of culture medium as the negative control. Clinical parameters, including respiratory disease scores and rectal temperature, were recorded daily from days −2 to 27 postinoculation, and blood samples were collected at 0, 3, 5, 7, 10, 14, and 21 dpi for viremia and serology analyses. Also, pigs were weighed at 0, 7, and 14 dpi to calculate the average daily weight gain (ADWG) (49, 50). Necropsy, gross and microscopic pathological evaluations, and immunohistochemistry (IHC) examinations were carried out once pigs died during the experiment, and all surviving pigs were euthanized and necropsied at 28 dpi as previously described (49, 50).

In parallel, the pathogenicity of viruses reversely passaged *in vitro* was also evaluated using the same processes, by using another batch of 25 pigs in 5 groups, PAM-R-25, PAM-R-60, JXwn06, JXwn06-P80, and the negative control.

### Genomic sequencing analysis of the reversely passaged PRRSV strains.

RT-PCR amplification and full-length genomic sequencing of PIG-R-3, PIG-R-5, PIG-R-9, PIG-R-10, PIG-R-11, PAM-R-25, PAM-R-60, and JXwn06-P80 were performed as previously described (26, 51, 52). Next, their complete genome sequences were aligned with those of JXwn06 and JXwn06-P80 by using Clustal W in Lasergene software (DNASTAR Inc., Madison, WI). The deduced amino acid sequences of different ORFs were also compared pairwise. The sequence of JXwn06 was set as the reference for nucleotide and amino acid positions.

### Construction of the virus with 4 reverse mutations in the genome of JXwn06-P80.

The full-length cDNA clone of JXwn06-P80 (pCMV-JXP80) was first constructed by using the vector pWSK29-CMV-MCS-HDV-SV40 according to a strategy similar to the one used in our previous report (Fig. 9A). Next, four revertant amino acid mutations found in the *in vitro* reversely passaged strain PAM-R-60 (Table 3) were introduced into the backbone plasmid pCMV-JXP80, which was then named pCMV-JXP80-4BM, and these two full-length plasmids were transfected into MARC-145 cells with Lipofectamine LTX reagent (Invitrogen, CA, USA) to rescue the viruses (47). After being identified by an indirect immunofluorescence assay (IFA) with anti-N mAb (SDOW17; Rural Technologies), the rescued viruses were named RvJXP80 and RvJXP80-4BM, respectively.

### Statistical analysis.

Data analysis was performed in GraphPad Prism version 5.0 (GraphPad Software, USA). Statistical significance between two groups was analyzed by using two-way analysis of variance (ANOVA) followed by a Bonferroni posttest. A two-tailed unpaired *t* test with Welch’s correction was used to estimate the significance of the gross lesion, histopathological, and immunohistochemistry scores of the lungs. Error bars represent the standard deviations (SD), as indicated in the figure legends. Differences were considered statistically significant at a *P* value of <0.05 and extremely significant at a *P* value of *<*0.01 or *<*0.001.

### Ethics statements.

All animal experiments were conducted according to the guidelines of the *Chinese Regulations of Laboratory Animals—the Guidelines for the Care of Laboratory Animals* (53) and *Laboratory Animal-Requirements of Environment and Housing Facilities* (54). All protocols for primary PAM preparation and animal inoculation were approved by the Laboratory Animal Ethical Committee of CAU, with approval no. AW07068102-2.

### Data availability.

The PRRSV strains mentioned in this study are available in GenBank under accession no. KP179402 for NT1, KP179403 for NT2, KP179404 for NT3, EF641008 for JXwn06, AY032626 for CH-1a, EU807840 for CH-1R, FJ548853 for JXA1-P80, EF536003 for VR-2332, and AF066183 for RespPRRS MLV.
