# Supplementary material for: Attenuated Porcine Reproductive and Respiratory Syndrome Virus Regains Its Fatal Virulence by Serial Passaging in Pigs or Porcine Alveolar Macrophages To Increase Its Adaptation to Target Cells
Source: Microbiol Spectr. 2022 Oct 11;10(6):e03084-22. doi: 10.1128/spectrum.03084-22 (PMC9769833; doi:10.1128/spectrum.03084-22)
Supplement: Supplemental file 1 — Tables S1 and S2. Download spectrum.03084-22-s0001.pdf, PDF file, 0.2 MB [file spectrum.03084-22-s0001.pdf]

Table S1. Genetic mutations occurred during the reversely passage *in vivo*.

		Nucleotide position	Amino acid position	JXwn06	JXwn06 -P80	PIG-R-3	PIG-R-5	PIG-R-9	PIG-R-10	PIG-R-11
ORF1a	NSP1a	336	/[*]	A	A	G	G	G	G	G
		535	/	C	C	T	T	T	T	T
	NSP1b	895	56	G(D)[#]	G(D)	A(N)	G(D)	G(D)	G(D)	G(D)
		951	74	G(E)	G(E)	T(D)	T(D)	T(D)	T(D)	T(D)
		974	82	C(A)	C(A)	C(A)	T(V)	C(A)	C(A)	C(A)
		1093	122	C(P)	C(P)	C(P)	C(P)	C(P)	G(A)	G(A)
	NSP2	1382	15	T(M)	T(M)	T(M)	T(M)	T(M)	C(T)	C(T)
		2124	/	C	C	C	C	C	T	C
		2348	337	G(C)	G(C)	G(C)	G(C)	G(C)	G(C)	A(Y)
		2620	428	A(T)	A(T)	G(A)	G(A)	G(A)	G(A)	G(A)
		2769	/	T	C	T	T	T	T	T
		2972	545	C(A)	C(A)	T(V)	T(V)	T(V)	T(V)	T(V)
		3115	593	T(S)	T(S)	C(P)	C(P)	C(P)	C(P)	C(P)
		3645	/	G	G	A	A	A	A	A
		3657	/	T	T	A	A	A	A	A
		3707	790	C(S)	C(S)	T(L)	T(L)	T(L)	T(L)	T(L)
		4653	/	C	C	T	T	T	T	T
	NSP3	5131	99	A(S)	A(S)	A(S)	T(C)	T(C)	T(C)	T(C)
		5357	174	T(I)	T(I)	C(T)	C(T)	C(T)	C(T)	C(T)
	NSP4	6129	/	C	T	C	C	C	C	C
	NSP7	6726	/	C	C	C	T	T	T	T
		7121	142	C(P)	T(L)	C(P)	C(P)	C(P)	C(P)	C(P)
ORF1b	NSP9	7941	112	A(T)	A(T)	G(A)	G(A)	G(A)	G(A)	G(A)
		8430	275	G(V)	G(V)	A(I)	A(I)	A(I)	A(I)	A(I)
		8609	/	G	G	A	A	A	A	A
		9143	/	C	C	T	T	T	T	T
		9203	/	T	T	T	T	T	C	C
		9509	/	A	A	G	G	G	G	G
	NSP10	9764	/	G	G	G	G	G	A	G
		10325	/	C	C	C	C	C	T	T
		10475	/	C	C	C	C	C	T	T
		10595	/	A	A	A	A	A	G	G
	NSP11	10895	/	C	C	T	T	T	T	T
		11090	/	T	T	C	T	C	C	C
	NSP12	11880	121	C(R)	T(W)	T(W)	C(R)	C(R)	C(R)	C(R)
ORF3	GP3	12728	/	C	C	C	C	C	T	T
		13033	143	T(F)	C(S)	C(S)	T(F)	T(F)	T(F)	T(F)
ORF4	GP4	13378	/	C	C	T	C	T	T	T
ORF5	GP5	13753	19	G(C)	G(C)	G(C)	G(C)	G(C)	A(Y)	A(Y)

	13936	80	G(G)	T(V)	T(V)	C(A)	G(G)	G(G)	G(G)

[*] This is in UTR or there is no amino acid change, only the nucleotide was shown;

[#] Nucleotide (amino acid).

Table S2. Genetic mutations occurred during the reversely passage *in vitro*.

		Nucleotide position	Amino acid position	JXwn06	JXwn06-P80	PAM-R-25	PAM-R-60
5'UTR		106	/[*]	C	C	C	T
		119	/	G	-[&]	-	-
ORF1a	NSP1a	535	/	C	C	T	C
		697	170	C(P)[#]	C(P)	T(S)	T(S)
	NSP1b	919	244	A(T)	A(T)	A(T)	G(A)
		956	256	A(Q)	A(Q)	G(R)	G(R)
		1047	/	A	A	G	G
	NSP2	2015	226	A(K)	A(K)	G(R)	G(R)
		3653	772	T(I)	T(I)	C(T)	C(T)
		3659	774	C(P)	C(P)	C(P)	T(L)
		4170	/	T	T	C	C
		4665	/	C	C	C	T
	NSP3	4850	5	T(I)	T(I)	T(I)	C(T)
	NSP4	5712	62	A(K)	A(K)	C(N)	C(N)
		6129	/	C	T	C	C
	NSP7	6766	24	T(F)	T(F)	C(L)	C(L)
		7199	168	G(R)	G(R)	A(K)	A(K)
		7324	210	A(T)	A(T)	A(T)	C(P)
	NSP8	7479	/	C	C	T	T
ORF1b	NSP9	8430	275	G(V)	G(V)	A(I)	A(I)
		8483	/	G	G	T	G
	NSP10	9563	/	C	C	A	A
		9680	/	T	T	C	C
		9853	109	G(R)	G(R)	A(K)	A(K)
	NSP12	11880	121	C(R)	T(N)	T(N)	A(R)
ORF2b	E	12012	9	G(D)	C(H)	C(H)	G(D)
ORF2a	GP2	12271	97	G(V)	G(V)	G(V)	T(L)
		12731	250	C(T)	C(T)	T(I)	T(I)
		12749	256	G(W)	G(W)	G(W)	A(stop)[$]
ORF4	GP4	13246	/	G	G	G	A
		13451	101	T(S)	T(S)	T(S)	G(A)
ORF5	GP5	13741	15	T(L)	A(H)	T(L)	T(L)
		13806	37	T(S)	T(S)	C(P)	C(P)
		14135	/	G	G	G	T

		14262	189	T(L)	G(V)	T(L)	T(L)
ORF7	N	14904	36	T(S)	T(S)	A(T)	A(T)
	N	15063	89	A(T)	A(T)	G(A)	G(A)

* This is in UTR or there is no amino acid change, only the nucleotide was shown;

& Nucleotide deletion;

Nucleotide (amino acid);

$ Stop codon (UGA).
